# Spatio-temporal stable isotope variation of a benthic primary consumer in a simple food web in a strongly acidic lake

**DOI:** 10.7717/peerj.7890

**Published:** 2019-10-11

**Authors:** Hideyuki Doi, Eisuke Kikuchi, Shigeto Takagi, Shuichi Shikano

**Affiliations:** 1 Graduate School of Life Sciences, Tohoku University, Sendai, Japan; 2 Graduate School of Simulation Studies, University of Hyogo, Kobe, Japan; 3 Center for Northeast Asian Studies, Tohoku University, Sendai, Japan; 4 Research Institute for Teacher Training and Development, Miyagi University of Education, Sendai, Japan

**Keywords:** Diatoms, Carbon and nitrogen stable isotopes, Chironomid, Phytoplankton, Lake Katanuma

## Abstract

Analysis of aquatic food webs is typically undertaken using carbon and nitrogen stable isotope composition of consumer and producer species. However, the trophic consequences of spatio-temporal variation in the isotope composition of consumers have not been well evaluated. Lake Katanuma, Japan, is highly acidic and has only one dominant species of benthic alga and one planktonic microalga, making it a prime system for studying trophic relationships between primary consumers and producers. In this simple lake food web, we conducted a field survey to evaluate spatial and temporal variation in the carbon and nitrogen stable isotope composition of a chironomid larvae in association with a single benthic and planktonic alga. We found a significant correlation between carbon stable isotope ratios of the chironomid larvae and the benthic diatom species in the lake. Thus, chironomid larvae may represent a reliable isotopic baseline for estimating isotope values in benthic diatoms. However, although the correlation held in shallow water, at four m depths, there was no significant relationship between the isotope ratios of chironomids and benthic diatoms, probably because deep-water larvae spend part of their life cycle migrating from the lake shore to deeper water. The differing isotope ratios of deeper chironomid tissues likely reflect the feeding history of individuals during this migration.

## Introduction

In aquatic ecosystems, the carbon and nitrogen stable isotope ratios of primary producers vary widely across time and space with both environmental and physiological factor changes (Zohary et al., 1994; Doi et al., 2003, 2004; Finlay, 2004). Thus, isotope composition of primary consumers that feed on primary producers may also vary temporally and spatially (Zohary et al., 1994; Finlay, Power & Cabana, 1999; Grey, Kelly & Jones, 2004; Frossard et al., 2013). Understanding variation in the baseline isotope values of both primary producers and primary consumers is essential for food web studies (Post, 2002). Variation in isotope values of consumer species reflects changes in diets and is influenced by many other factors, including the time-lag between producer and baseline isotope values and the migration behavior of baseline species (Post, 2002; Gustafson et al., 2007). Also, the isotopic values of consumers such as bivalves and snails often reflected the isotopic values of primary producers (Post, 2002; Gustafson et al., 2007). Therefore, our understanding of spatio-temporal isotopic variation in consumer taxa is important for isotope studies. However, the available data on isotopic variability remains limited, especially in natural systems.

Microalgae are important primary producers in many freshwater ecosystems (Wetzel, 2001). Generally, microalgal isotope ratios are measured for an entire algal community, for example, as particulate organic matter (POM; mainly phytoplankton) or benthic algal mats (Bootsma et al., 1996; Yoshii, 1999). For lake food webs, such autochthonous source as well as the allochthonous sources such as terrestrial litters are important sources (Marcarelli et al., 2011). The pooling of tens or hundreds of species blurs patterns of spatial and temporal variability and the difficulty associated with the separation of each microalgal species prevents the measurement of species-specific isotope ratios. Pooling of this type precludes proper comparisons of isotope ratios between primary producer and consumer species because the effects of the content and form of each algal species on consumer feeding must be considered.

The acidic waters of Lake Katanuma (mean pH 2.2), Japan, support only two species of microalgae: the benthic diatom *Pinnularia acidojaponica* and the planktonic green alga *Chlamydomonas acidophila*. In this lake, there is only one consumer species, a deposit-feeding chironomid larva (*Chironomus acerbiphilus*) that feeds mainly on benthic diatoms in shallow waters compared with potential food sources, such as terrestrial supply (Doi, Kikuchi & Shikano, 2001; Doi et al., 2006). For lake food webs, such autochthonous source as well as the allochthonous sources such as terrestrial litters are important sources (Marcarelli et al., 2011). Also, the methane bacteria may be the source (Jones et al., 2008; Grey, 2016); however, methane production in the lake would be very limited due to the highly acidic water. The simplicity of this three-species food web provides a unique opportunity to analyze the temporal and spatial variations in isotope signatures of consumer species. In this study, we investigate the nature and reason for isotopic changes in the chironomid larvae within the unique Lake Katanuma system.

## Materials and Methods

### Sampling and sample preparation

The study was conducted in Lake Katanuma (38°44′N, 140°43′E), a water body of volcanic origin located in northeastern Japan. There are no inflowing or outflowing streams, and the lake is surrounded by oak forest and sand beach. The lake surface area is 0.14 km^2^, and the maximum depth is 20 m. Oak litter supply into the lake is scarce due to the limited forest area and the absence of inflowing streams (Doi et al., 2003).

Larvae of the chironomid *Chironomus acerbiphilus*, benthic diatoms, sediment, and water samples for POM (predominantly *Chlamydomonas acidophila*; see Doi et al., 2003, 2004) analysis were collected in November and December 2000, and in June, July, October, November, and December 2001. We were unable to collect samples in the months without chironomid larvae, as dissolved oxygen concentrations decreased to undetectable levels throughout the whole water column following hydrogen sulfide release from the lake bottom in late July 2001 (Shikano et al., 2004; Takagi et al., 2005; Takagi, 2006). We also did not collect samples when the lake was iced over (late December to March). The generation time of the chironomid was ~ a month, and the chironomid had four to five generation per year in the lake (Takagi et al., 2005), and the isotope turnover time was conformed less than 6 days from our feeding experiment (Doi et al., 2007). Samples were 3collected from three stations at different water depths (one, two, and four m), because *Chironomus acerbiphilus* almost inhabited <4 m depth (Takagi et al., 2005). *Chironomus acerbiphilus* larvae were collected in triplicate using an Ekman-Birge sediment sampler. In the laboratory, the larvae were sorted from sediment immediately and transferred into filtered lake water for at least 24 h to allow elimination of gut contents. The elimination method was confirmed by our previous studies (Doi et al., 2006, 2007). Animals were then freeze-dried for preservation. Up to three individuals of fourth-instar *Chironomus acerbiphilus* larvae comprised a sample.

The samples of a phytoplankton *Chironomus acerbiphilus* and a benthic alga *P*. *acidojaponica* were collected by filtering the lake water and separating from sediment using its phototactic movements, respectively, as previously described in Doi et al. (2003). Surface water samples for POM analysis were collected from the three stations with a Van-Dorn sampler (three L volume) at the time of *Chironomus acerbiphilus* collection. The collected waters were filtered through glass filters (Whatman GF/F, precombusted at 500 °C for 2 h) in order to collect POM samples. Sediment samples for *P. acidojaponica* separation were collected concurrently from the lake bottom with an Ekman-Birge grab at 0.5 cm at one, two, and four m depths. Cells of *P*. *acidojaponica* were separated from the sediment using its phototactic movements (Couch, 1989; Doi et al., 2003). Sediment was spread to a depth of one cm in a Petri dish, and a nylon net (75 µm mesh) was placed on the surface. The net was covered with a two mm layer of 400 µm particle diameter silica powder (pre-combusted at 500 °C for 2 h). Petri dishes were illuminated for 24 h, while moisture was maintained with continuous spraying of filtered lake water onto the silica. After illumination, the silica powder containing *P. acidojaponica* was scraped off and mixed with filtered deionized water. Suspended *P. acidojaponica* were poured into glass vials and freeze-dried. Samples were preserved by freezing at −20 °C until analysis of stable isotope ratios was performed. The contribution of plant litter to the chironomid larval diet was minimal, and any litter assimilated may be regarded as a subsidiary food (Doi et al., 2006). Thus, we did not consider terrestrial plant litter as a potential food source for the chironomid larvae. The isotope values of the species and POM contained some missing data, because we could not collect the individuals and the samples due to their absence or lower density in the season and the depths (Takagi et al., 2005).

### Stable isotope analysis

The carbon and nitrogen isotope ratios of the samples were measured by mass spectrometry (Finnigan Mat; DELTA plus, Waltham, MA, USA). The results are reported using delta notation: δ^13^C or δ^15^N = (*R*_sample_/*R*_standard_ − 1) × 1,000 (‰), where *R* is the ^13^C/^12^C or ^15^N/^14^N ratio for δ^13^C or δ^15^N, respectively. As global standards, PeeDee Belemnite was used for δ^13^C and atmospheric nitrogen for δ^15^N. The analytical errors were within ±0.2‰ for both δ^13^C and δ^15^N. The δ^15^N data for benthic diatoms were limited since we were unable to collect sufficient samples for δ^15^N analysis. We could not measure the isotope signature of microbial productions, but we should note that the autotrophic production of the lake largely contributed to *Chironomus acerbiphilus* as based on our previous studies (Doi, Kikuchi & Shikano, 2001, 2006).

### Statistical analysis

We performed all statistical analyses using R ver. 3.5.1 software (R Development Core Team, 2008). We compared the δ^13^C and δ^15^N of benthic diatoms and chironomid larvae using cross-correlation and Pearson’s correlation coefficients by *ccf* and *cor.test* function in R. From cross-correlation, we found the time lag was not influenced the correlation for the both time series due to *r*_*t* = 0_ was the highest values among the time lag; 0.671–1.0. Because of sampling limitations, the correlation analysis for δ^15^N is based on a smaller dataset than equivalent analyses for δ^13^C.

## Results

**Figure 1:**
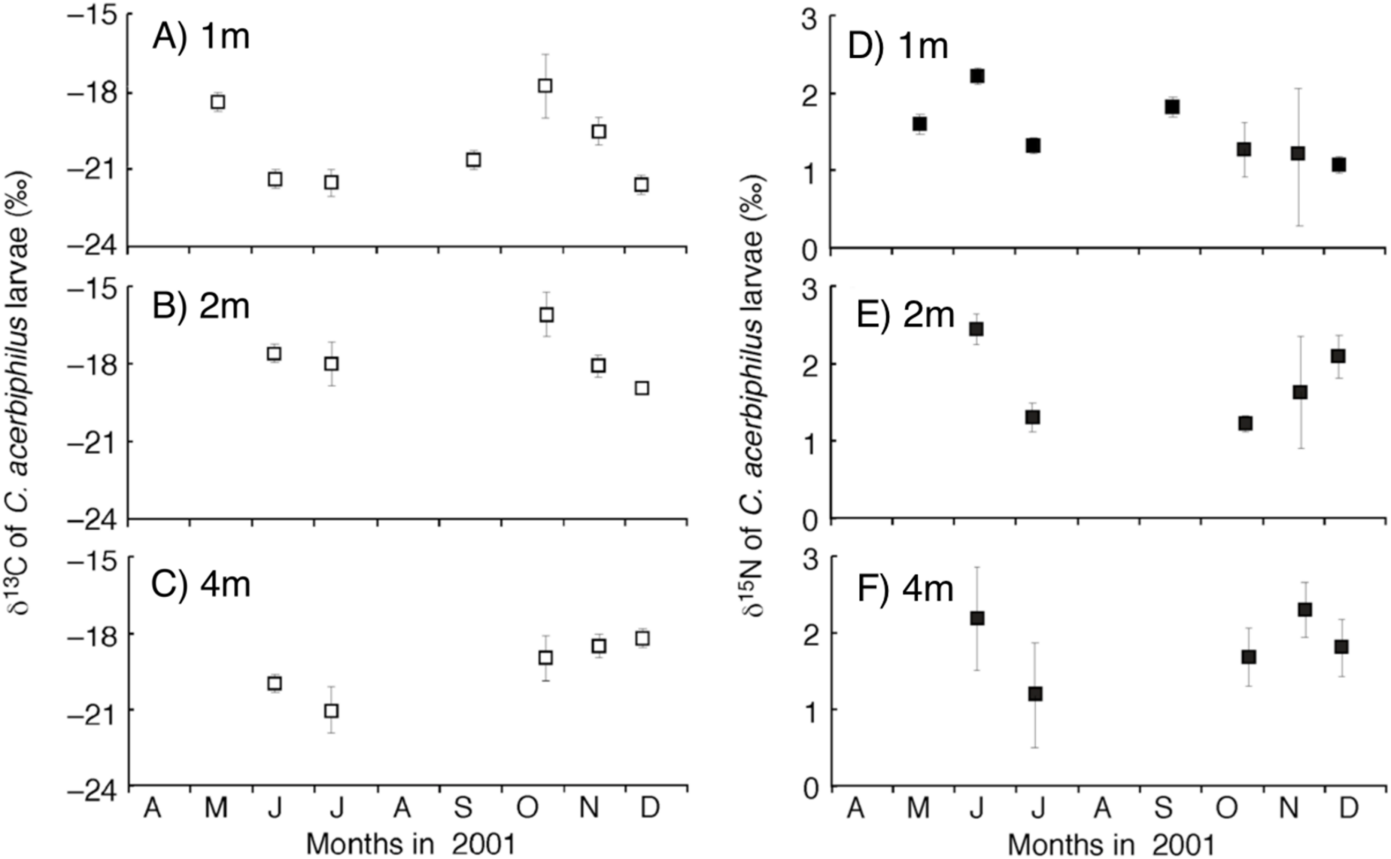
Temporal trend of carbon and nitrogen isotope values of *Chironomus acerbiphilus* larvae. (A–C) Carbon isotope values of Chironomus acerbiphilus larvae at 1-, 2- and 4-m water depth. (D–F) Nitrogen isotope values of Chironomus acerbiphilus larvae at 1-, 2- and 4-m water depth. Error bar means ± 1 SD (*N* = 3).

**Figure 2:**
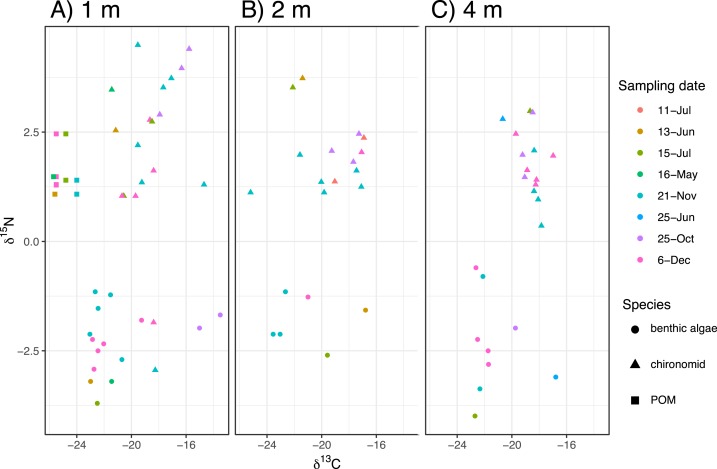
The biplot of carbon and nitrogen isotope values of the samples including the chironomid larvae (*Chironomus acerbiphilus*) and benthic diatoms (*Pinnularia acidojaponica*) and POM in the sampling depth and months. (A–C) Biplots at 1-, 2- and 4-m water depth.

**Figure 3:**
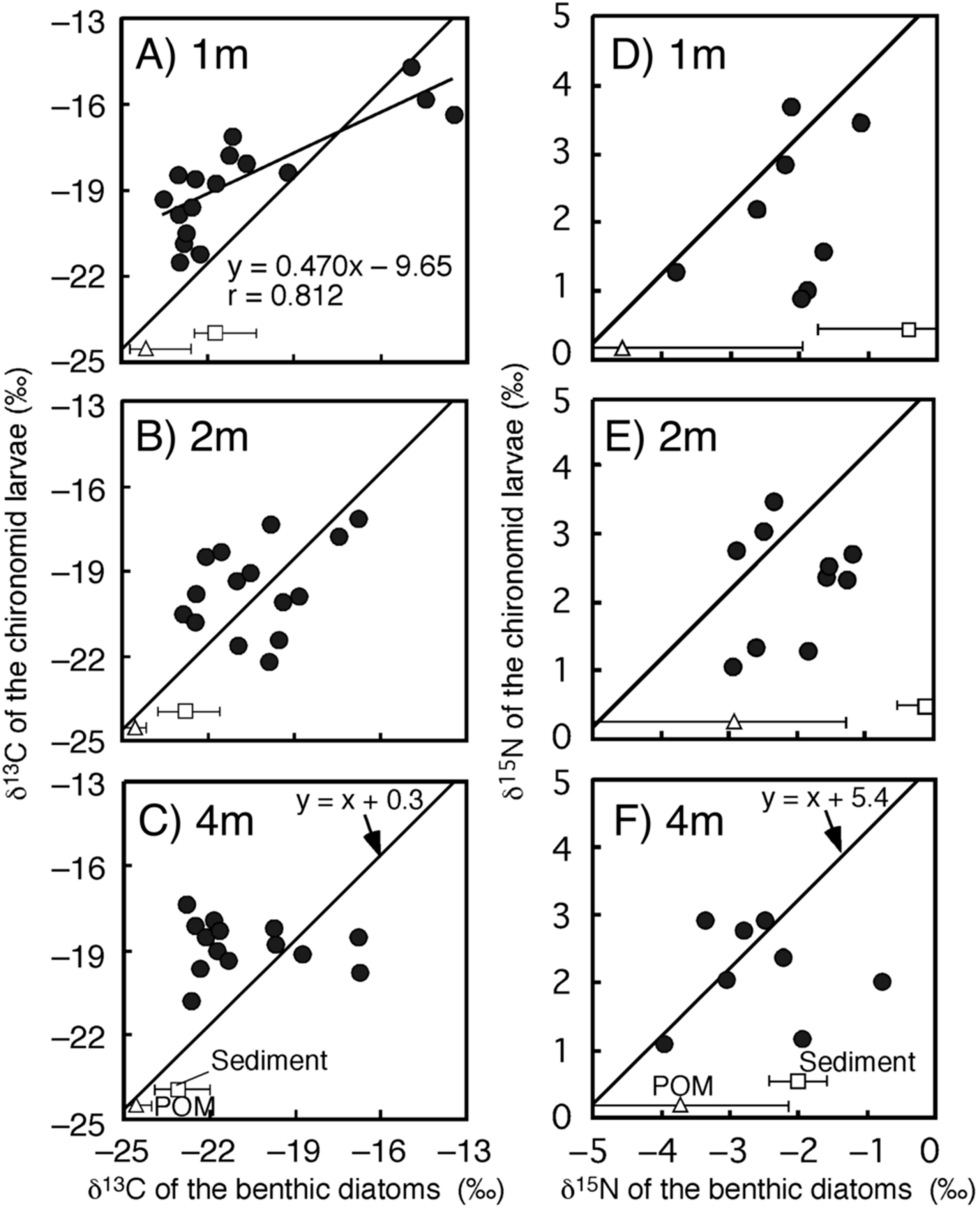
Relationships between isotope values of the chironomid larvae (*Chironomus acerbiphilus*) and benthic diatoms (*Pinnularia acidojaponica*) at each water depth. (A–C) Carbon isotope values at 1-, 2- and 4-m water depth. (D–F) Nitrogen isotope values at 1-, 2- and 4-m water depth. The lines were *y* = *x* + 0.3 for C and *x* + 5.4 for N using the specific isotope fractionation of the chironomid from Doi et al. (2006). Triangle and square plots indicate the mean isotope values of POM and sediment at each water depth. The error bar means ± 1 SD (*N* = 3).

The time-series of the δ^13^C and δ^15^N values of the benthic diatoms and that of the chironomid larvae were shown in Figs. 1A–1F. The seasonal variations in chironomid larvae and the producers were found in the Figs. 2 and 3. In some cases, δ^13^C values of benthic diatoms were higher than those of the chironomid larvae, though the consumers generally have little higher δ^13^C values compared to their diets. This may be caused by temporal fluctuation in δ^13^C values of the benthic diatom as well as the differences in the turnover times between them. There was a significant positive relationship between the δ^13^C of the benthic diatoms and that of the chironomid larvae at one m depth (Pearson’s correlation coefficient, *r* = 0.812, *p* = 0.001, *n* = 18, Fig. 3A). We can therefore assume that in this time period, the chironomid larvae fed mainly on benthic diatoms. There was a weak positive relationship between the δ^13^C values of the benthic diatoms and the chironomid larvae at two m water depth (*r* = 0.465, *p* = 0.09, *n* = 15, Fig. 3B), while no significant relationship was observed between the benthic diatoms and the chironomid larvae from the four m water depth (*r* = −0.152, *p* = 0.681, *n* = 14, Fig. 3C).

There was no significant relationship between δ^15^N values of chironomid larvae and benthic diatoms at any depth (one m: *r* = 0.337, *p* = 0.210, two m: *r* = 0.192, *p* = 0.378, four m: *r* = 0.022, *p* = 0.950, Figs. 3D–3F). In all cases, however, the sample size was small (*n* = 8–10 for the correlation). The mean differences in δ^15^N values between chironomid larvae and benthic diatoms were 4.7 ± 1.1‰, which is similar to the laboratory isotope fractionation values for benthic diatoms and phytoplankton (5.4 ± 1.2‰, mean ± SD; Doi et al., 2006).

## Discussion

We observed changes in the relationship between the isotopic values of chironomid larvae and their food sources at different water depths in the lake. The δ^13^C values of chironomid larvae were largely different from those of sediment and POM, thus, the chironomid larvae may be considered as an isotope baseline reflecting the temporal and spatial variation in isotope values of benthic diatoms (Doi, Kikuchi & Shikano, 2001; Frossard et al., 2013). Indeed, at depths of one and two m, there were strong correlations between δ^13^C of the chironomid and benthic diatoms. In Lake Katanuma, *P. acidojaponica* is the only benthic diatom. Thus, the isotope values of chironomid larvae might reflect isotope changes in a single microalgal species. The isotope change in *P. acidojaponica* is caused by boundary layer effects, which are considerable in such small benthic organisms (Doi et al., 2003). This could indicate that the isotope values of such primary consumers may be useful in estimating temporal and spatial variation in the isotope values of benthic primary producers.

In early-instar stages, the time from the initial isotope measurement to 50% turnover in the chironomid was 6 days, and during late-instar stages, the isotope turnover changed only slightly (Doi et al., 2007). While, for the fourth instars, the isotopic values of the chironomid reflects those of the benthic diatoms over at least 1 week. Further, a meta-analysis study (Vander Zanden et al., 2015) provided a linear model to predict the species-specific half-life time of isotope turnover using the organisms’ body size. From a model for the relationships between half-life days of isotope turnover and invertebrate body size (ln(isotope half-life day) = 0.23 * ln(body size) + 3.25), which was provided by a meta-analysis, the isotopic half-life for the chironomid can be predicted 3.1 days (the body size: ~0.6 g from Doi et al. (2007)). Therefore, we could assume that the isotope turnover of the chironomid larvae were relative very short. In previous studies, long-lived consumers such as bivalves and snails were considered to be good isotope baselines for long-lived consumers such as fish (Post, 2002). In this study of short-lived animals (chironomids) with rapid isotope turnover, we demonstrated clear isotopic relationships between the chironomid larvae and benthic diatoms. Thus, such a short-lived primary consumer may be useful in estimating trophic level and food sources in other short-lived consumer species. Woodland et al. (2012) developed the dynamic mixing model to evaluate the relative contribution of benthic vs. pelagic carbon sources with considering the baseline dynamics. The approach may help to understand the temporal dynamics in the resource contribution to a consumer. While, for our study species, the life cycle of the chironomid was less than a month (Takagi et al., 2005). Due to the life cycle might be shorter than our sampling interval, we cannot apply the dynamic mixing model in this study. Future study needs to consider the sampling interval and the life cycle to considering the resource baseline dynamics.

At four m water depth of this lake, the correlation between isotopic ratios of the chironomid and benthic diatoms was not significant. This lack of correlation at four m likely reflects chironomid migration from the lakeshore to the lake bottom. The eggs of *Chironomus acerbiphilus* are laid on the lakeshore, and the larvae at four m depth have mostly immigrated from the shore into the deeper lake waters (Takagi, 2006). Thus, the isotope signatures of the chironomid larvae at four m depth might track their migration across the depth gradient.

Motile animals usually display spatial variation in their isotope ratios, the magnitude and direction of which varies between different ecosystems depending on biogeochemical processes (DeNiro & Epstein, 1978; Webster et al., 2002; Wissel & Fry, 2005). Because of this spatial heterogeneity in isotope signatures, animals migrating between isotopically distinct ecosystems record within their tissues isotopic information from their previous feeding locations (Tieszen & Boutton, 1988; Hobson & Clark, 1992). Accordingly, for establishing isotope baselines, it is essential (for migratory species) to take into account migration tracks between habitats that differ in primary producer isotope signatures.

## Conclusions

In summary, we found that at the one and two m water depths, there was a significant correlation between isotope signatures of chironomid larvae and benthic diatoms. The chironomid larvae may be considered a good isotope baseline species for tracking isotope values of benthic diatoms. This study conducted with the simplicity of this three-species food web in Lake Katanuma for the temporal and spatial variations in isotope signatures of consumer species. This correlative relationship held true across a range of spatio-temporal variability in the diatoms. However, the correlation of samples taken at a depth of four m was not significant, probably due to migration behaviors of *Chironomus acerbiphilus*. Further study needs to consider the migration, feeding and other behaviors to consider the changes in the correlation between the isotope signatures of chironomid larvae and benthic diatoms.

## Supplemental Information

10.7717/peerj.7890/supp-1 Supplemental Information 1 The raw data of the samples. The dataset showed carbon (δ^13^C) and nitrogen (δ^15^N) stable isotope data (unit: ‰) of the samples in this study.

## Additional Information and Declarations

### Data Availability

The following information was supplied regarding data availability: The raw measurements are available as a Supplemental File.

## References

[ref-1] Bootsma HA, Hecky RE, Hesslein RH, Turner GF (1996). Food partitioning among lake Malawi nearshore fishes as revealed by stable isotope analysis. Ecology.

[ref-2] Couch CA (1989). Carbon and nitrogen stable isotopes of meiobenthos and their food resources. Estuarine, Coastal and Shelf Science.

[ref-3] DeNiro MJ, Epstein S (1978). Influence of diet on the distribution of carbon isotopes in animals. Geochimica et Cosmochimica Acta.

[ref-4] Doi H, Kikuchi E, Hino S, Itoh T, Takagi S, Shikano S (2003). Seasonal dynamics of carbon stable isotope ratios of particulate organic matter and benthic diatoms in strongly acidic Lake Katanuma. Aquatic Microbial Ecology.

[ref-5] Doi H, Kikuchi E, Shikano S (2001). Carbon and nitrogen stable isotope ratios analysis of food sources for *Chironomus acerbiphilus* larvae(Diptera Chironomidae) in strongly acidic Lake Katanuma. Radioisotopes.

[ref-6] Doi H, Kikuchi E, Shikano S, Takagi S (2004). A study of the nitrogen stable isotope dynamics of phytoplankton in a simple natural ecosystem. Aquatic Microbial Ecology.

[ref-7] Doi H, Kikuchi E, Takagi S, Shikano S (2006). Selective assimilation by deposit feeders: experimental evidence using stable isotope ratios. Basic and Applied Ecology.

[ref-8] Doi H, Kikuchi E, Takagi S, Shikano S (2007). Changes in carbon and nitrogen stable isotopes of chironomid larvae during growth, starvation and metamorphosis. Rapid Communications in Mass Spectrometry.

[ref-9] Finlay JC (2004). Patterns and controls of lotic algal stable carbon isotope ratios. Limnology and Oceanography.

[ref-10] Finlay JC, Power ME, Cabana G (1999). Effects of water velocity on algal carbon isotope ratios: implications for river food web studies. Limnology and Oceanography.

[ref-11] Frossard V, Belle S, Verneaux V, Millet L, Magny M (2013). A study of the δ^13^C offset between chironomid larvae and their exuvial head capsules: implications for palaeoecology. Journal of Paleolimnology.

[ref-12] Grey J (2016). The incredible lightness of being methane-fuelled: stable isotopes reveal alternative energy pathways in aquatic ecosystems and beyond. Frontiers in Ecology and Evolution.

[ref-13] Grey J, Kelly A, Jones RI (2004). High intraspecific variability in carbon and nitrogen stable isotope ratios of lake chironomid larvae. Limnology and Oceanography.

[ref-14] Gustafson L, Showers W, Kwak T, Levine J, Stoskopf M (2007). Temporal and spatial variability in stable isotope compositions of a freshwater mussel: implications for biomonitoring and ecological studies. Oecologia.

[ref-15] Hobson KA, Clark RG (1992). Assessing avian diets using stable isotopes I: turnover of ^13^C in tissues. Condor.

[ref-16] Jones RI, Carter CE, Kelly A, Ward S, Kelly DJ, Grey J (2008). Widespread contribution of methane-cycle bacteria to the diets of lake profundal chironomid larvae. Ecology.

[ref-28] Marcarelli AM, Baxter CV, Mineau MM, Hall RO (2011). Quantity and quality: unifying food web and ecosystem perspectives on the role of resource subsidies in freshwaters. Ecology.

[ref-17] Post DM (2002). Using stable isotopes to estimate trophic position: models methods, and assumptions. Ecology.

[ref-18] R Development Core Team (2008). R: a language and environment for statistical computing.

[ref-19] Shikano S, Kikuchi E, Takagi S, Do H (2004). Volcanic heat flux and short-term holomixis during the summer stratifloation period in crater lake. Limnology and Oceanography.

[ref-20] Takagi S (2006). Population dynamics and secondary production of *Chironomus acerbiphilus* (Diptera: Chironomidae) in the strongly acidic Lake Katanuma.

[ref-21] Takagi S, Kikuchi E, Doi H, Shikano S (2005). Swimming behaviour of *Chironomus acerbiphilus* larvae in Lake Katanuma. Hydrobiologia.

[ref-22] Tieszen LL, Boutton TW, Rundel PW, Ehleringer JR, Nagy NA (1988). Stable carbon isotopes terrestrial ecosystem research. Stable Isotopes in Ecological Research.

[ref-29] Vander Zanden MJ, Clayton MK, Moody EK, Solomon CT, Weidel BC (2015). Stable isotope turnover and half-life in animal tissues: a literature synthesis. PLOS ONE.

[ref-23] Webster MS, Marra PP, Haig SM, Bensch S, Holmes RT (2002). Links between worlds: unraveling migratory connectivity. Trends in Ecology & Evolution.

[ref-27] Wetzel RG (2001). Limnology: lake and river ecosystems.

[ref-24] Wissel B, Fry B (2005). Tracing Mississippi River influences in estuarine food webs of coastal Louisiana. Oecologia.

[ref-30] Woodland RJ, Rodríguez MA, Magnan P, Glémet H, Cabana G (2012). Incorporating temporally dynamic baselines in isotopic mixing models. Ecology.

[ref-25] Yoshii K (1999). Stable isotope analyses of benthic organisms in Lake Baikal. Hydrobiologia.

[ref-26] Zohary T, Erez J, Gophen M, Berman-Frank I, Stiller M (1994). Seasonality of stable carbon isotopes within the pelagic food web of Lake Kinneret. Limnology and Oceanography.

